# Single- versus double-layer closure of the caesarean (uterine) scar in the prevention of gynaecological symptoms in relation to niche development – the 2Close study: a multicentre randomised controlled trial

**DOI:** 10.1186/s12884-019-2221-y

**Published:** 2019-03-04

**Authors:** S. I. Stegwee, I. P. M. Jordans, L. F. van der Voet, M. Y. Bongers, C. J. M. de Groot, C. B. Lambalk, R. A. de Leeuw, W. J. K. Hehenkamp, P. M. van de Ven, J. E. Bosmans, E. Pajkrt, E. A. Bakkum, C. M. Radder, M. Hemelaar, W. M. van Baal, H. Visser, J. O. E. H. van Laar, H. A. A. M. van Vliet, R. J. P. Rijnders, M. Sueters, C. A. H. Janssen, W. Hermes, A. H. Feitsma, K. Kapiteijn, H. C. J. Scheepers, J. Langenveld, K. de Boer, S. F. P. J. Coppus, D. H. Schippers, A. L. M. Oei, M. Kaplan, D. N. M. Papatsonis, L. H. M. de Vleeschouwer, E. van Beek, M. N. Bekker, A. J. M. Huisjes, W. J. Meijer, K. L. Deurloo, E. M. A. Boormans, H. W. F. van Eijndhoven, J. A. F. Huirne

**Affiliations:** 1 Department of Obstetrics and Gynaecology, Research institutes ‘Amsterdam Cardiovascular Sciences’ and ‘Amsterdam Reproduction and Development’, Amsterdam UMC, Vrije Universiteit Amsterdam, De Boelelaan 1117, 1081 HV Amsterdam, Netherlands; 2 Department of Obstetrics and Gynaecology, Deventer Hospital, Nico Bolkesteinlaan 75, 7416 SE Deventer, the Netherlands; 3 Department of Obstetrics and Gynaecology, Máxima Medical Centre, De Run 4600, 5504 DB Veldhoven, the Netherlands; 4 Department of Epidemiology and Biostatistics, Vrije Universiteit Amsterdam, De Boelelaan 1105, 1081 HV Amsterdam, the Netherlands; 5 Department of Health sciences, Faculty of Science, Research institute ‘Amsterdam Public Health’, Vrije Universiteit Amsterdam, De Boelelaan 1085, 1081 HV Amsterdam, the Netherlands; 6 Department of Obstetrics and Gynaecology, Amsterdam UMC, University of Amsterdam, Meibergdreef 9, 1105 AZ Amsterdam, the Netherlands; 7 Department of Obstetrics and Gynaecology, OLVG-oost, Oosterpark 9, 1091 AC Amsterdam, the Netherlands; 8 Department of Obstetrics and Gynaecology, OLVG-west, Jan Tooropstraat 164, 1061 AE Amsterdam, the Netherlands; 9 Department of Obstetrics and Gynaecology, Westfriesgasthuis, Maelsonstraat 3, 1624 NP Hoorn, the Netherlands; 10 Department of Obstetrics and Gynaecology, Flevo hospital, Hospitaalweg 1, 1315 RA Almere, the Netherlands; 11 Department of Obstetrics and Gynaecology, Tergooi hospital, Rijksstraatweg 1, 1261 AN Blaricum, the Netherlands; 12 Department of Obstetrics and Gynaecology, Catharina hospital, Michelangelolaan 2, 5623 EJ Eindhoven, the Netherlands; 13 Department of Obstetrics and Gynaecology, Jeroen Bosch hospital, Henri Dunantstraat 1, 5223 GZ ‘s-Hertogenbosch, the Netherlands; 14 Department of Obstetrics and Gynaecology, Leiden University Medical Centre, Albinusdreef 2, 2333 ZA Leiden, the Netherlands; 15 Department of Obstetrics and Gynaecology, Groene Hart hospital, Bleulandweg 10, 2803 HH Gouda, the Netherlands; 16 Department of Obstetrics and Gynaecology, Haaglanden Medical Centre – Westeinde hospital, Lijnbaan 32, 2512 VA Den Haag, the Netherlands; 17 Department of Obstetrics and Gynaecology, Haga hospital, Els-Borst-Eilersplein 275, 2545 AA Den Haag, the Netherlands; 18 Department of Obstetrics and Gynaecology, Reinier de Graaf hospital, Reinier de Graafweg 5, 2625 AD Delft, the Netherlands; 19 Department of Obstetrics and Gynaecology, Research school ‘GROW’, Maastricht University Medical Centre, P. Debyelaan 25, 6229 HX Maastricht, the Netherlands; 20 Department of Obstetrics and Gynaecology, Zuyderland Medical Centre, Henri Dunantstraat 5, 6419 PC Heerlen, the Netherlands; 21 Department of Obstetrics and Gynaecology, Rijnstate hospital, Wagnerlaan 55, 6815 AD Arnhem, the Netherlands; 22 Department of Obstetrics and Gynaecology, Radboud University Nijmegen Medical Centre, Geert Grooteplein Zuid 10, 6525 GA Nijmegen, the Netherlands; 23 Department of Obstetrics and Gynaecology, Canisius-Wilhelmina hospital, Weg door Jonkerbos 100, 6532 SZ Nijmegen, the Netherlands; 24 Department of Obstetrics and Gynaecology, Bernhoven hospital, Nistelrodeseweg 10, 5406 PT Uden, the Netherlands; 25 Department of Obstetrics and Gynaecology, Röpcke-Zweers hospital, Jan Weitkamplaan 4a, 7772 SE Hardenberg, the Netherlands; 26 Department of Obstetrics and Gynaecology, Amphia hospital, Langendijk 75, 4819 EV Breda, the Netherlands; 27 Department of Obstetrics and Gynaecology, Sint Franciscus hospital, Kleiweg 500, 3045 PM Rotterdam, the Netherlands; 28 Department of Obstetrics and Gynaecology, Sint Antonius hospital, Koekoekslaan 1, 3435 CM Nieuwegein, the Netherlands; 29 Department of Obstetrics and Gynaecology, Birth Centre Wilhelmina Children hospital/University Medical Centre Utrecht, Lundlaan 6, 3584 EA Utrecht, the Netherlands; 30 Department of Obstetrics and Gynaecology, Gelre hospital – location Apeldoorn, Albert Schweitzerlaan 31, 7334 DZ Apeldoorn, the Netherlands; 31 Department of Obstetrics and Gynaecology, Gelre hospital – location Zutphen, Den Elterweg 77, 7207 AE Zutphen, the Netherlands; 32 Department of Obstetrics and Gynaecology, Diakonessenhuis, Bosboomstraat 1, 3582 KE Utrecht, the Netherlands; 33 Department of Obstetrics and Gynaecology, Meander Medical Centre, Maatweg 3, 3813 TZ Amersfoort, the Netherlands; 34 Department of Obstetrics and Gynaecology, Isala clinics, Dokter van Heesweg 2, 8025 AB Zwolle, the Netherlands

**Keywords:** Caesarean section, Closure techniques, Long-term outcomes, Postmenstrual spotting, Niche, Quality of life, Fertility, Reproductive outcomes

## Abstract

**Background:**

Double-layer compared to single-layer closure of the uterus after a caesarean section (CS) leads to a thicker myometrial layer at the site of the CS scar, also called residual myometrium thickness (RMT). It possibly decreases the development of a niche, which is an interruption of the myometrium at the site of the uterine scar. Thin RMT and a niche are associated with gynaecological symptoms, obstetric complications in a subsequent pregnancy and delivery and possibly with subfertility.

**Methods:**

Women undergoing a first CS regardless of the gestational age will be asked to participate in this multicentre, double blinded randomised controlled trial (RCT). They will be randomised to single-layer closure or double-layer closure of the uterine incision. Single-layer closure (control group) is performed with a continuous running, unlocked suture, with or without endometrial saving technique. Double-layer closure (intervention group) is performed with the first layer in a continuous unlocked suture including the endometrial layer and the second layer is also continuous unlocked and imbricates the first. The primary outcome is the reported number of days with postmenstrual spotting during one menstrual cycle nine months after CS. Secondary outcomes include surgical data, ultrasound evaluation at three months, menstrual pattern, dysmenorrhea, quality of life, and sexual function at nine months. Structured transvaginal ultrasound (TVUS) evaluation is performed to assess the uterine scar and if necessary saline infusion sonohysterography (SIS) or gel instillation sonohysterography (GIS) will be added to the examination. Women and ultrasound examiners will be blinded for allocation. Reproductive outcomes at three years follow-up including fertility, mode of delivery and complications in subsequent deliveries will be studied as well. Analyses will be performed by intention to treat. 2290 women have to be randomised to show a reduction of 15% in the mean number of spotting days. Additionally, a cost-effectiveness analysis will be performed from a societal perspective.

**Discussion:**

This RCT will provide insight in the outcomes of single- compared to double-layer closure technique after CS, including postmenstrual spotting and subfertility in relation to niche development measured by ultrasound.

**Trial registration:**

Dutch Trial Register (NTR5480). Registered 29 October 2015.

**Electronic supplementary material:**

The online version of this article (10.1186/s12884-019-2221-y) contains supplementary material, which is available to authorized users.

## Background

Caesarean section (CS) rates have increased from 14.5 to 27.2% in the last two decades in the Western world. [1] In 2016, 26.664 CSs were performed in the Netherlands, being 16.0% of the total number of deliveries. [2] The increasing CS rate has stimulated an interest in the potential long-term morbidity of a CS scar, such as uterine rupture or malplacentation. [3–7] Other less severe, but more prevalent long-term symptoms are gynaecological symptoms and subfertility.

Only recently, gynaecological symptoms such as painful menstruations and postmenstrual spotting have been associated with CSs. [8–10] These symptoms are considered to be related to a niche, defined as *“an indentation at the site of the caesarean scar with a depth of at least 2 mm”*, visible on transvaginal ultrasound (TVUS). [11] Two cohort studies reported a strong association between postmenstrual spotting and a niche: odds ratio (OR) 3.1; 95% confidence interval (CI) 1.5–6.3 [8] and OR 5.5; 95% CI 1.1–26.5. [10] In these studies, a niche was observed in 50 to 60% of the women after a CS, using transvaginal ultrasound. [8, 10] Spotting was correlated to niche volume and inversely correlated to the residual myometrium thickness (RMT). [8, 10]

In addition to the gynaecological symptoms, a niche may influence fertility. A recent meta-analysis reported that a CS on average reduced the probability of subsequent pregnancy with 9% (relative risk (RR) 0.91; 95% CI 0.87–0.95) in comparison to a vaginal delivery. [12] None of the included studies in this meta-analysis evaluated the relation between subsequent fertility and the presence of a niche. One of the hypotheses is that intra-uterine fluid or cervical mucus or blood accumulation in the niche are expected to hamper the penetration of sperm cells or impair embryo implantation. [13] Long-term follow-up will facilitate the evaluation of the association between uterine closure, niche development, accumulation of intra-uterine fluid and subfertility.

In the last years, various therapies have been developed and implemented to treat niche related symptoms such as menstrual disorders. [14–18] Effectiveness of both hysteroscopic [19] and laparoscopic niche resection [15] have recently been published. Because both niche related symptoms and applied therapies lead to increases in medical consultations and costs, it seems to be more efficient to prevent niche development in the first place. Uterine closure technique of the CS scar has been proposed as an independent factor for niche development. [9] However, large randomised trials evaluating the effect of uterine closuring techniques on postmenstrual spotting or other gynaecological or reproductive outcomes in relation to niche development and thin residual myometrium are lacking, as well as cost-effectiveness evaluations.

In order to shorten surgery time and in the absence of significant differences in short-term outcomes [20, 21], most Dutch gynaecologists (92%) have replaced double-layer by single-layer closure after a CS, using multifilament continuous unlocked sutures. Given the higher risk on myometrium loss and thus development of a thinner residual myometrium after single-layer closure [5, 22], we hypothesise that this method introduces a higher risk on postmenstrual spotting and possibly subfertility after a CS and that it can be prevented by applying double-layer unlocked closure.

Double-layer unlocked closure is considered safe, without a clinically relevant higher risk on short-term outcomes. [5, 23, 24]. Moreover, it results in a thicker residual myometrium, especially when unlocked sutures are applied. [5, 22, 24] Dysmenorrhea was reported more frequently after single-layer closure, but this was only studied in two RCTs and not always related to ultrasound findings such as myometrial thickness or niche presence. [24] Prevalence of uterine rupture seems to be similar after single- versus double-layer closure [5, 22, 24], but has neither been related to ultrasound findings and since it has a very low incidence, statistically significant differences are difficult to find. Since long-term outcomes such as gynaecological symptoms, fertility outcomes and results of subsequent pregnancies are studied infrequently, additional evidence is needed before a preference for either technique can be indicated.

## Objective

Our primary objective is to determine the effectiveness of unlocked double-layer uterine closure compared to unlocked single-layer uterine closure in the prevention of niche related gynaecological symptoms nine months after a first CS. Secondary objectives are to assess niche prevalence measured by ultrasound at three months follow-up and to study both reproductive outcomes related to a subsequent pregnancy and gynaecological symptoms at three years follow-up. Additionally we aim to study the cost-effectiveness alongside the trial.

## Methods/design

### Design

This multicentre randomised controlled superiority trial will be performed in the Netherlands, in hospitals that collaborate within the Dutch Consortium for Healthcare Evaluation and Research in Obstetrics and Gynaecology (NVOG Consortium 2.0, www.zorgevaluatienederland.nl). Centres that participate are district, teaching or university hospitals in the Netherlands. A list of study sites is available in Additional file 1.

### Participants and eligibility criteria

All women who undergo a first CS, planned or unplanned, will be asked to participate in the study. Other inclusion criteria are: sufficient command of the Dutch or English language, age ≥ 18 years and written informed consent. To prevent confounding effects on niche development during the study, we will exclude women with a previous CS. Other exclusion criteria are: inadequate possibility for counselling (e.g. indication for emergency CS without being informed about the study previously, women in severe pain without adequate therapy), previous major uterine surgery (e.g. laparoscopic or laparotomic fibroid resection, septum resection), women with known causes of menstrual disorders (e.g. cervical dysplasia, communicating hydrosalpinx, uterine anomaly or endocrine disorders disturbing ovulation), placenta in- or percreta during the current pregnancy or ≥ three foetuses during the current pregnancy.

### Recruitment and randomisation

Eligible women will be asked by a gynaecologist, resident, clinical midwife or research nurse to participate in the trial when they undergo a planned CS. Eligible women who are planned to undergo a vaginal delivery will also be informed about this study during pregnancy in case they need an unplanned CS. Furthermore, women during induced labour and women receiving adequate therapy for pain during labour, will be asked to participate in case a CS is needed during labour for any indication.

**Fig. 1:**
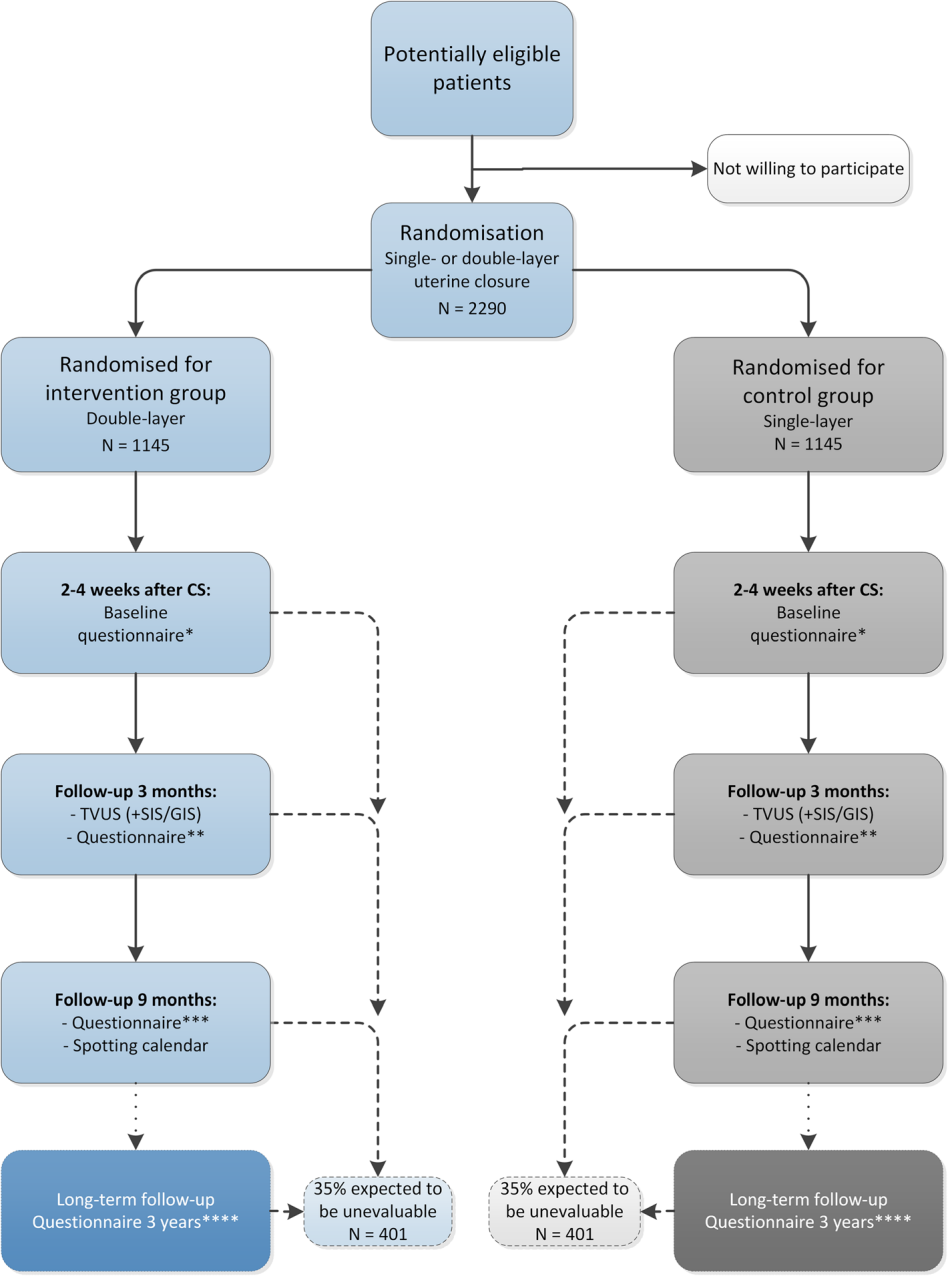
Flowchart of the 2Close study. * = baseline questions, EQ-5D-5L. ** = symptom questionnaire, EQ-5D-5L, SF36, PROMIS SF8a, iMCQ, iPCQ. *** = symptom questionnaire, FSFI, EQ-5D-5L, SF36, iMCQ, iPCQ. **** = symptom questionnaire, fertility questionnaire, FSFI, EQ-5D-5L, SF36, PROMIS SF8a

When the decision of a CS is made and all selection criteria are met, women will be randomly allocated to single-layer (control group) or double-layer (intervention group) closure (1:1) (see Fig. 1). Randomisation will be performed using a web-based application ALEA 2.2 which displays a computer-generated random number, managed by the Clinical Research Unit of the Amsterdam UMC - location AMC. We will use a permuted block-design, stratified for recruiting centres and for planned or unplanned CS. All women that decline to participate will be registered anonymously in order to record the number and reason for refusal. Subjects who withdraw from this study will not be replaced. 

Gynaecologists, residents, clinical midwives or research nurses enrol participants and assign them to the intervention. The CS will be performed by either a gynaecologist, a resident supervised by a gynaecologist or by a resident that is authorised to perform CSs without supervision. Participants and sonographers will be blinded for the closure technique. If operative reintervention after CS is needed and the gynaecologist that performs the reintervention needs to know the closure technique that the participant was assigned to, unblinding is possible through the logistic trial coordinator. We expect this situation to occur very infrequently.

### Intervention (double-layer closure)

**Fig. 2:**
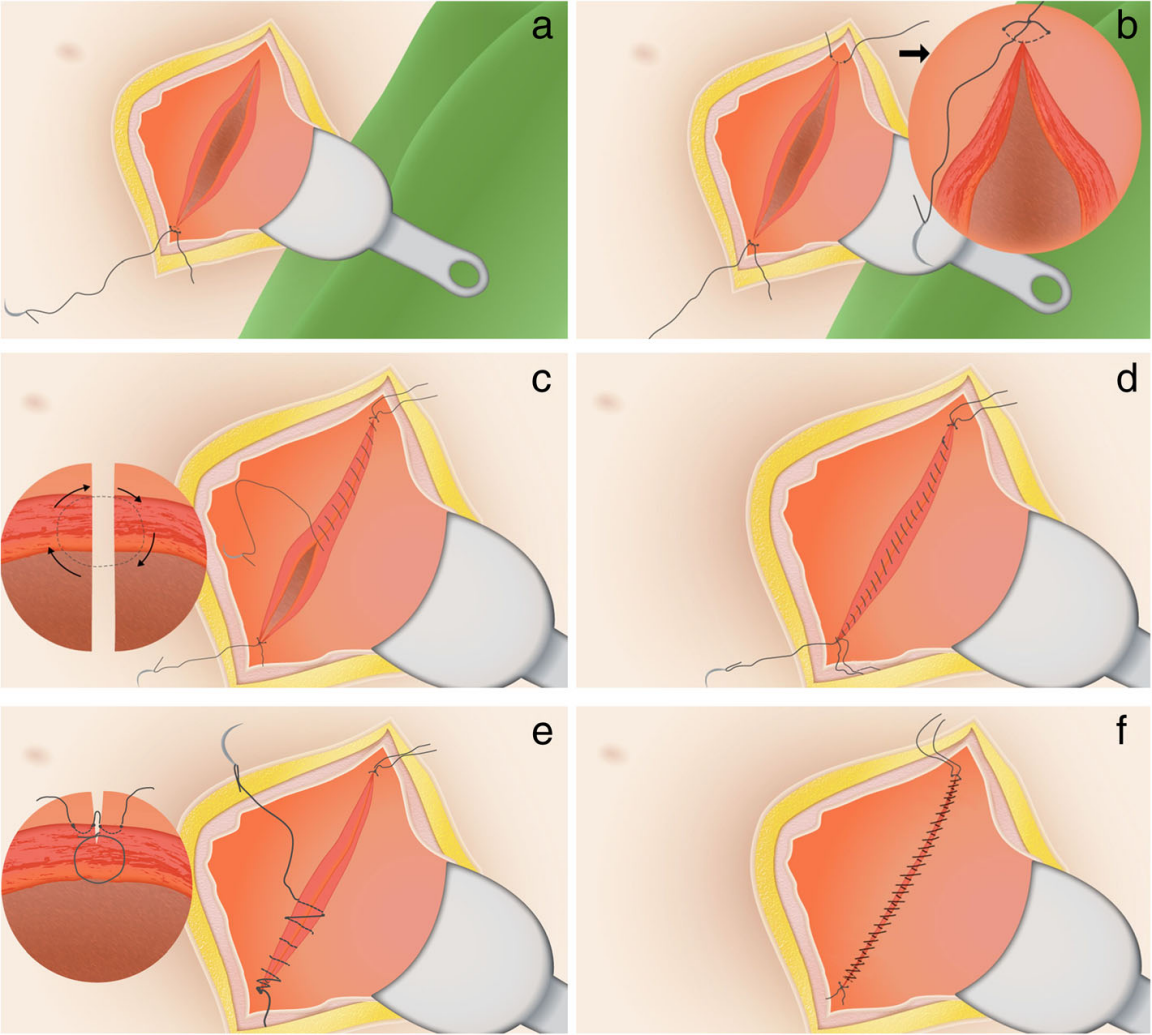
Double-layer uterine closure technique. **a**. Step 1: lateral suture; **b**. Step 2: lateral suture on the other side; **c**. Step 3: First layer: full thickness, continuous, including large part of myometrium, including the endometrial layer; **d**. Step 4: End of this first layer; **e**. Step 5: Second layer: superficial continuous layer of serosal tissue, imbricating the first layer; **f**. Step 6: First and second layer should be closely connected

In both study arms, women will undergo a CS following a standard way with respect to mode of uterotomy, correct approximations of the cutting edges and non-closure of the peritoneum. In the intervention arm, double-layer closure of the uterus will be performed using unlocked multifilament continuous running sutures for both layers and the endometrial layer will be included in the first layer (see Fig. 2). The second layer is a continuous running suture that imbricates the first layer. Since this is not the standard method for uterine closure in the Netherlands, a short online instruction film will be shown to all participating centres and surgeons prior to participation (see Additional file 2). Surgical outcomes will be registered after the procedure in the electronic case report form (eCRF).

### Control group (single-layer closure)

The control group will receive usual closure technique of the uterus: a single-layer closure using unlocked continuous running multifilament sutures. The currently available evidence is inconclusive with respect to endometrial saving technique or not. Therefore, we decided that in our study surgeons are free to choose to close either full thickness (including the endometrium) or split thickness (excluding the endometrium) in the control group. The applied method, including endometrial saving technique or not, will be registered.

### Niche evaluation

**Fig. 3:**
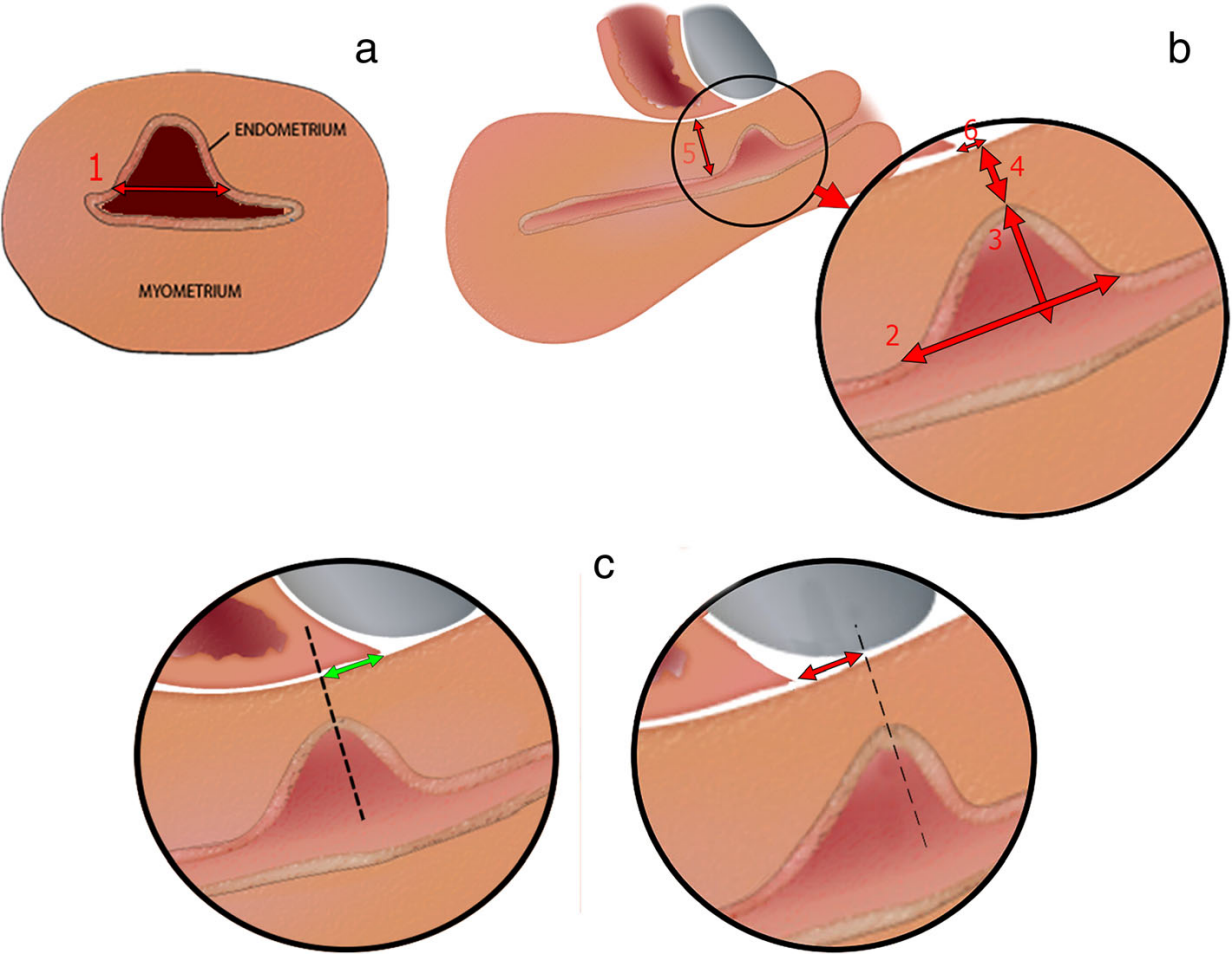
Standardised transvaginal ultrasound evaluation of a niche in the non-pregnant uterus. **a**. Measured in the transversal plane: niche width (1); **b**. Measured in the sagittal plane: niche length (2), niche depth (from cervical canal until apex of the niche) (3), residual myometrium thickness (from deepest part of the niche until the serosa) (4), adjacent myometrium thickness (myometrium thickness close to base of the niche) (5), distance from apex of the niche to vesico-vaginal fold (see **c**) (6); **c**. Measuring the niche relatively to the vesico-vaginal fold in the sagittal plane: positive value (green arrow, in mm) or negative value (red arrow, in mm)

The care after CS will be according to the normal local protocol with the regular outpatient visit that is normally executed six weeks after the CS. This routine visit may be postponed to three months after the CS to enable an ultrasound evaluation to identify the existence of a niche, but participating centres may decide whether they want visits at six weeks (routine follow-up) and at three months (ultrasound follow-up) or only one visit after three months combining the regular control and the ultrasound follow-up. The ultrasound evaluation is standardised as proposed by Jordans et al. [11] (see Fig. 3). Based on this standardisation, we created an obligatory e-learning for all ultrasound performers to let all ultrasounds be performed in a uniform manner. To increase consistency and to improve the learning curve, we will evaluate a sample of ultrasounds in each centre based on recorded pictures and provide feedback to the examiners. Since it is known that a niche can be missed during TVUS only [8, 10, 25] we will additionally perform a saline infusion sonohysterography (SIS) or gel installation sonography (GIS) in case no niche is observed during the normal TVUS or if the ultrasound is inconclusive. It would be optimal to have a contrast enhanced ultrasound in all women when the uterine cavity or niche are not naturally filled with fluid, but we have chosen for this approach to prevent unnecessary burden for the participants and to reduce costs.

### Outcome measures

#### Primary outcome measure

The primary outcome is the number of days of postmenstrual spotting during one cycle at nine months after CS. We defined postmenstrual spotting as brownish discharge for more than two days at the end of the menstruation, with a total duration (menstruation and spotting) of more than seven days, or intermenstrual blood loss that starts after the end of the menstruation. [8] The number of days of postmenstrual spotting will be counted as follows: days with brownish discharge (> two days) when the total duration of menstruation and spotting exceeds seven days + number of days with intermenstrual blood loss. Amenorrhoeic women, due to lactation, medication or other diseases, will not be evaluable for the primary outcome and will be left out of this analysis.

#### Secondary outcome measures at short-term

- Perioperative outcomes including blood loss, operative time, additional haemostatic sutures and complications.
- Menstruation characteristics, dysmenorrhea (visual analogue scale (VAS)), Quality of Life (QOL) using Short-Form-36 [26] and EQ-5D-5L [27, 28], societal reintegration (PROMIS Short-Form-8a [29]), sexual function using the Female Sexual Function Index (FSFI [30]), applied medical and/or surgical therapy because of gynaecological symptoms, all obtained through digital questionnaires, will be assessed at three and nine months follow-up.
- Ultrasound evaluation will be performed at three months follow-up using TVUS, in which RMT, adjacent myometrium thickness (AMT), presence of a niche (depth of ≥2mm), length, depth and width of the niche, presence of large niches (RMT < 50% of AMT, RMT <3mm) and niche volume will be measured.

#### Cost-effectiveness outcomes

Costs will be measured using adapted versions of the iMTA Productivity Cost Questionnaire (iPCQ [31]) and iMTA Medical Consumption Questionnaire (iMCQ [32]) from a health care and societal perspective at nine months of follow-up.

#### Secondary outcome measures at long-term

- Menstruation characteristics, pain, sexual functioning, QOL and social reintegration will be evaluated at three years follow-up.
- Reproductive outcomes at three years follow-up: % of women desiring to conceive, % of women that conceived including time to conceive, % of women with an ongoing pregnancy, the need for fertility treatment and pregnancy outcomes such as mode of delivery or complications will be determined.

Long-term outcomes will be presented in a separate article.

### Data collection and data management

#### Intraoperative data

Immediately after the CS we will register relevant items regarding the delivery and CS in an eCRF, in which confidentiality and anonymity are ensured and audit trails are accessible. These items include: reason for planned or unplanned CS, emergency CS or not, whether women experienced contractions, dilatation, performed method for uterine closure, endometrial saving technique applied, used suturing material, extra haemostatic sutures, operative time, blood loss and complications.

#### Collection of baseline characteristics and patient reported outcomes

Baseline characteristics will be collected through a digital questionnaire at 2–4 weeks after caesarean section, sent to the e-mail address of participants. Since we will also include unplanned CSs, we decided that it is not possible for all participants to answer questions regarding baseline characteristics before the operation. Baseline parameters include maternal age, body mass index, social economic status, smoking habit, medical and obstetric history, gestational age and previous vaginal deliveries, all reported by the participant. We expect that the impact of niche related symptoms such as postmenstrual spotting on daily activities and sexual behaviour may be influenced by ethnic background and religion, therefore we will also register these characteristics. At three months, nine months and three years follow-up, again digital questionnaires will be sent to participants to assess the primary and secondary outcomes (see Fig. 1). At nine months, we ask participants record their exact menstrual and spotting pattern, if any, in an adjusted menstruation score chart. [33] Reminders for all questionnaires will be sent every two weeks, with a maximum of three times. When no response is given after the reminders, research nurses from participating centres will be asked to call the participant.

#### Data niche evaluation

Results of the TVUS and GIS or SIS, performed three months after CS, will be registered. Women will not receive information regarding the presence of a niche, since it has no clinical consequences so shortly after CS and this may influence the answers given in the questionnaires. Other important abnormalities visualised by ultrasound will be reported as usual.

### Statistical issues

#### Sample size calculation

We use a superiority design since we expect double-layer closure to be favourable. Literature for making reliable estimations on postmenstrual spotting in relation to niches is scarce. We have used baseline data from the HysNiche [19] and LapNiche [15] study. We estimate the mean number of spotting days to be 3.5 days/month in the total group. We consider a 15% reduction in the mean number of spotting days clinically relevant, which is 0.5 day/month reduction. Assuming a standard deviation (SD) of 3.4 and a two-sided significance level of 5%, a total of 1488 women need to be included to achieve a power of 80%. Increasing the sample size to take into account 35% of women unevaluable (due to drop-out, non-response or amenorrhoea) for the primary outcome, 2290 women need to be included.

#### Data-analysis

Data-analysis will be performed according to the intention to treat principle and additional per protocol analyses will be performed. A test will be considered statistically significant when the two-sided test shows a *p*-value < 0.05. Baseline characteristics will be presented using percentages, means with SD and 95% CI or medians with interquartile ranges (IQR), where appropriate.

The primary outcome, number of days of postmenstrual spotting, will be presented for both groups as mean with SD or median with IQR, and presented in a Box-Whisker graph to show the distribution. Differences in primary outcome between the groups will be tested using the independent t-test in case of normal distribution (possible after transformation of the outcome) or Mann-Whitney U test. An adjusted analysis will be performed using linear regression analysis in which we adjust for factors on which randomisation was stratified and for baseline factors on which relevant differences are observed despite randomisation.

Dichotomous secondary outcomes will be presented as percentages and RR with corresponding 95% CI. *P*-values will be calculated using the chi-square test or, if the expected count for at least one cell is below 5, using the Fisher exact test. Normally distributed continuous variables will be presented as means with SD, and differences between the groups will be calculated with an independent t-test. Non-normally distributed continuous variables will be presented as medians with IQR and differences between the groups will be calculated with Mann-Whitney U test. The questionnaires will be analysed using the appropriate algorithms and usual presentation methods (FSFI, EQ-5D-5L, SF36, PROMIS SF8a, iMCQ, iPCQ).

Comparison of primary outcome between women receiving single- and double-layer closure will be done as secondary analyses within each of the following subgroups separately:

1. Planned (without labour) or unplanned (in labour) CS
2. Emergency CS or not
3. Preterm (< 37 weeks gestational age) or term (≥ 37 week gestational age) CS
4. Presence (> 3cm) or absence (≤ 3cm) of dilatation
5. Placenta praevia or not
6. Presence or absence of specific maternal morbidity (e.g. diabetes, pre-eclampsia, haemolysis/elevated liver enzymes/low platelet count (HELLP) syndrome, immunodeficient women)
7. Singleton versus twin pregnancy
8. Natural cycle or hormonally induced withdrawal bleeding

Within the single-layer group (control group) we will compare the primary outcome between women in whom endometrial saving technique (split thickness) was applied and women in whom an endometrial saving technique was not applied (full thickness).

#### Economic evaluation

The economic evaluation will be performed alongside the RCT from a societal perspective. Both a cost-effectiveness and cost-utility analysis will be performed with a time horizon of nine months to relate the difference in societal and healthcare costs between double-layer and single-layer unlocked uterine closure during a CS to the difference in clinical effects. Healthcare costs include costs of primary and secondary care, complementary care and home care. Costs in other sectors include presence and absence from paid and unpaid work. The friction cost approach will be used to estimate indirect costs. For the valuation of health care utilization standard prices published in the Dutch Costing guidelines will be used. [34] Medication use will be valued using prices of the Royal Dutch Society for Pharmacy.

Societal costs will be related to the following effect measures in the economic evaluation: days with postmenstrual spotting and quality-adjusted life-years (QALYs) based on the Dutch tariff for the EuroQol (EQ-5D-5L). [27, 28, 35]

We hypothesise that double-layer uterine closure will reduce postmenstrual spotting and related consultations for gynaecological or fertility related problems and applied therapies, and as a consequence that it will be cost-effective in comparison with single-layer uterine closure.

The analysis will be done according to the intention to treat principle. Missing costs and effect data will be imputed using multiple imputation. Incremental cost-effectiveness ratios (ICERs) will be calculated by dividing the difference in mean total costs between the treatment groups by the difference in mean effects. Bootstrapping with 5000 replications will be used to estimate 95% CI around cost differences and the uncertainty surrounding the ICERs. Uncertainty surrounding the ICERs will be graphically presented on cost-effectiveness planes. Cost-effectiveness acceptability curves showing the probability that double-layer uterine closure is cost-effective in comparison with single-layer uterine closure for a range of different ceiling ratios will also be estimated. Adjustment for confounders and effect modifiers will be done if necessary. [36]

#### Interim analysis and safety monitoring

Because of the type of intervention, the Medical Ethics Committee (MEC) determined that the risk for participation is negligible. Therefore, we do not have a Data Safety Monitoring Committee. No interim analysis is planned.

All serious adverse events (SAEs) will be reported to the MEC by line listing yearly. Life threatening SAEs or an event that leads to death will be reported to the MEC immediately. All SAEs will be followed until they have abated, until a stable situation has been reached or the patient was discharged. We do not expect to terminate the study prematurely given the low risk of adverse events.

#### Confidentiality and data security

All participating centres receive a login name and password to gain access to ALEA 2.2, the web-secured randomisation database. Randomisation is performed pseudo-anonymously with only the initials and year of birth of the participants. Linking personal data to the study number can only be performed in the local participating centres or by the trial coordinator (SS). Written informed consent forms are stored in every centre in a lockable room. All forms and data will be archived for 15 years in the participating centres.

## Discussion

In the last years, studies examining complications of CSs are increasing, including the development of niches or thin residual myometrium at the site of the previous CS and related symptoms. Both RMT and the presence of a niche have been associated with postmenstrual spotting. [8, 10] Double-layer unlocked closure has been shown to result in a thicker residual myometrium and as a consequence can possibly lead to a decrease of niche development after a CS compared to single-layer closure. [5, 22, 24, 37] However, the long-term clinical outcomes in terms of postmenstrual spotting or subfertility have not been studied previously or have not been related to ultrasound findings. We hypothesise that niche related postmenstrual spotting and fertility problems will reduce together with decrease in niche prevalence, in which identification of the best uterine closure technique regarding RMT and niche development will be of great significance.

### Strengths and limitations

The design of this study is one of the strengths; this is the first large RCT that will evaluate the effectiveness of double-layer uterine closure compared to single-layer uterine closure after CS regarding niche related gynaecological symptoms and reproductive outcomes with a long-term follow-up. The study is adequately powered. Randomisation is performed by using a web based randomisation program. Furthermore, all participants and examiners are blinded which reduces the chance for bias regarding reported symptoms and ultrasound findings. An additional strength is the uniform manner in which we try to perform double-layer closure and ultrasound evaluation, instructed by mandatory online instruction film and e-learning, respectively. Moreover, the 2Close study will compare the cost-effectiveness of both techniques which has never been done before. As we expect that double-layer closure will reduce the incidence of niche development and as a consequence that it could possibly reduce the gynaecological symptoms including postmenstrual spotting after CS, we assume double-layer closure to be more cost-effective. Also, we expect that double-layer closure will improve the chances of conceiving after CS and lower costs in fertility treatment.

We also expect some limitations. Baseline characteristics will be collected through questionnaires that are filled in by women in the first month after CS, which might lead to recall bias regarding medical history, complications during pregnancy and labour, and other baseline measurements. We decided to lower the administrative load for participating hospitals by obtaining these characteristics through the participants. Furthermore, there is no validated questionnaire available yet for postmenstrual spotting; therefore, the questionnaires that are used in the 2Close study are not adjusted or validated for these symptoms. Moreover, the surgical techniques performed during the CS in this study are standardised in both study arms except for saving the endometrium in the control group. There is no conclusive evidence whether or not to save the endometrium in the suture according to its influence on niche development. Therefore, we chose to leave this decision with the surgeons. There may possibly be a difference in the incidence of niche development between the participants receiving single-layer split thickness or full thickness closure, also when compared to the incidence of niche development in the double-layer group. This will be further examined in a subgroup analysis.

To prevent bias regarding niche evaluation three months after CS, all ultrasonographic examiners are trained by an online learning program and a sample of ultrasounds will be evaluated. The learning module is based on the results of a Delphi procedure among international niche experts. [11] Although the niche examiners in the 2Close are trained by a standardised method, experience in measuring niches and as a consequence differences in niche measurement may occur among examiners.

### Potential impact and implications

This study will gain insight in the most optimal uterine closure technique after CS which is relevant for women and gynaecologists, since we will focus on long-term gynaecological symptoms and reproductive outcomes in relation to changes of the lower uterine segment after CS and in particular niche development. Since many studies have already shown that RMT and niches are related to several symptoms and therapies for niche resection are being developed, we think it is necessary to provide evidence for the development of preventive strategies regarding niche related symptoms. It is important to realise that the best way to prevent a niche and its related symptoms, is to not perform a CS. But since it is often inevitable to perform a CS, care takers should perform it in the most optimal way.

After the results of this study become available, the most optimal and cost-effective technique can be implemented in order to reduce symptoms and problems in a subsequent pregnancy. This will not be difficult, since the technique is easy to learn and many gynaecologists and residents are familiar with it after the trial. Especially for a scheduled CS, women should be informed about the risk to develop a niche and the risk that it might cause symptoms or complications later in life.

## Additional files

Additional file 1: Affiliations of all 32 participating hospitals in the Netherlands that granted approval. The board of the hospitals granted approval to participate and to start recruiting patients. (DOCX 15 kb)

Additional file 2: Text of the online standardised instruction film for double-layer closure of the uterotomy. The spoken text in the online instruction film, which shows a standardised way to perform double-layer closure of the uterotomy, has been translated into English. (DOCX 15 kb)

## Abbreviations

AMT: Adjacent myometrium thickness
BROK: Basic course Regulations and Organisation for Clinical researchers
CI: Confidence interval
eCRF: electronic case report form
CS: Caesarean section
GCP: Good clinical practice
GIS: Gel instillation sonohysterography
ICER: Incremental cost-effectiveness ratio
iMCQ: iMTA Medical Consumption Questionnaire
iMTA: Institute for Medical Technology Assessment
iPCQ: iMTA Productivity Cost Questionnaire
IQR: Interquartile range
MEC: Medical ethics committee
OR: Odds ratio
QALY: Quality adjusted life years
QOL: Quality of life
RCT: Randomised controlled trial
RMT: Residual myometrium thickness
RR: Relative risk
SAE: Serious adverse event
SD: Standard deviation
SIS: Saline infusion sonohysterography
TVUS: Transvaginal ultrasound
VAS: Visual analogue scale
