# Supplementary material for: Single- versus double-layer closure of the caesarean (uterine) scar in the prevention of gynaecological symptoms in relation to niche development – the 2Close study: a multicentre randomised controlled trial
Source: BMC Pregnancy Childbirth. 2019 Mar 4;19:85. doi: 10.1186/s12884-019-2221-y (PMC6399840; doi:10.1186/s12884-019-2221-y)
Supplement: Supplementary file 1 — Affiliations of all 32 participating hospitals in the Netherlands that granted approval. The board of the hospitals granted approval to participate and to start recruiting patients. (DOCX 15 kb) [file 12884_2019_2221_MOESM1_ESM.docx]

**Additional file 1. Affiliations of participating hospitals that granted approval**

1. Amsterdam UMC, Vrije Universiteit Amsterdam, Department of Obstetrics and Gynaecology, De Boelelaan 1118, 1081 HV Amsterdam, the Netherlands.
2. Deventer Hospital, Department of Obstetrics and Gynaecology, Nico Bolkesteinlaan 75, 7416 SE Deventer, the Netherlands
3. Amsterdam UMC, University of Amsterdam, Department of Obstetrics and Gynaecology, Meibergdreef 9, 1105 AZ Amsterdam, the Netherlands
4. OLVG-oost, Department of Obstetrics and Gynaecology, Oosterpark 9, 1091 AC Amsterdam, the Netherlands
5. OLVG-west, Department of Obstetrics and Gynaecology, Jan Tooropstraat 164, 1061 AE Amsterdam, the Netherlands
6. Westfriesgasthuis, Department of Obstetrics and Gynaecology, Maelsonstraat 3, 1624 NP Hoorn, the Netherlands
7. Flevo hospital, Department of Obstetrics and Gynaecology, Hospitaalweg 1, 1315 RA Almere, the Netherlands
8. Tergooi hospital, Department of Obstetrics and Gynaecology, Rijksstraatweg 1, 1261 AN Blaricum, the Netherlands
9. Máxima Medical Centre, Department of Obstetrics and Gynaecology, De Run 4600, 5504 DB Veldhoven, the Netherlands
10. Catharina hospital, Department of Obstetrics and Gynaecology, Michelangelolaan 2, 5623 EJ Eindhoven, the Netherlands
11. Jeroen Bosch hospital, Department of Obstetrics and Gynaecology, Henri Dunantstraat 1, 5223 GZ 's-Hertogenbosch, the Netherlands
12. Leiden University Medical Centre, Department of Obstetrics and Gynaecology, Albinusdreef 2, 2333 ZA Leiden, the Netherlands
13. Groene Hart hospital, Department of Obstetrics and Gynaecology, Bleulandweg 10, 2803 HH Gouda, the Netherlands
14. Haaglanden Medical Centre – Westeinde hospital, Department of Obstetrics and Gynaecology, Lijnbaan 32, 2512 VA Den Haag, the Netherlands
15. Haga hospital, Department of Obstetrics and Gynaecology, Els-Borst-Eilersplein 275, 2545 AA Den Haag, the Netherlands
16. Reinier de Graaf hospital, Department of Obstetrics and Gynaecology, Reinier de Graafweg 5, 2625 AD Delft, the Netherlands
17. Maastricht University Medical Centre, Department of Obstetrics and Gynaecology, P. Debyelaan 25, 6229 HX Maastricht, the Netherlands
18. Zuyderland Medical Centre, Department of Obstetrics and Gynaecology, Henri Dunantstraat 5, 6419 PC Heerlen, the Netherlands
19. Rijnstate hospital, Department of Obstetrics and Gynaecology, Wagnerlaan 55, 6815 AD Arnhem, the Netherlands
20. Radboud University Nijmegen Medical Centre, Department of Obstetrics and Gynaecology, Geert Grooteplein Zuid 10, 6525 GA Nijmegen, the Netherlands
21. Canisius-Wilhelmina hospital, Department of Obstetrics and Gynaecology, Weg door Jonkerbos 100, 6532 SZ Nijmegen, the Netherlands
22. Bernhoven hospital, Department of Obstetrics and Gynaecology, Nistelrodeseweg 10, 5406 PT Uden, the Netherlands
23. Röpcke-Zweers hospital, Department of Obstetrics and Gynaecology, Jan Weitkamplaan 4 a, 7772 Hardenberg, the Netherlands
24. Amphia hospital, Department of Obstetrics and Gynaecology, Langendijk 75, 4819 EV Breda, the Netherlands
25. Sint Franciscus hospital, Department of Obstetrics and Gynaecology, Kleiweg 500, 3045 PM Rotterdam, the Netherlands
26. Sint Antonius hospital, Department of Obstetrics and Gynaecology, Koekoekslaan 1, 3435 CM Nieuwegein, the Netherlands
27. Birth Centre Wilhelmina Children hospital/University Medical Centre Utrecht, Department of Obstetrics and Gynaecology, Lundlaan 6, 3584 EA Utrecht, the Netherlands
28. Gelre hospital – location Apeldoorn, Department of Obstetrics and Gynaecology, Albert Schweitzerlaan 31, 7334 DZ Apeldoorn, the Netherlands
29. Gelre hospital – location Zutphen, Department of Obstetrics and Gynaecology, Den Elterweg 77, 7207 AE Zutphen, the Netherlands
30. Diakonessenhuis, Department of Obstetrics and Gynaecology, Bosboomstraat 1, 3582 KE Utrecht, the Netherlands
31. Meander Medical Centre, Department of Obstetrics and Gynaecology, Maatweg 3, 3813 TZ Amersfoort, the Netherlands
32. Isala clinics, Department of Obstetrics and Gynaecology, Dokter van Heesweg 2, 8025 AB Zwolle, the Netherlands
