# Supplementary material for: Single- versus double-layer closure of the caesarean (uterine) scar in the prevention of gynaecological symptoms in relation to niche development – the 2Close study: a multicentre randomised controlled trial
Source: BMC Pregnancy Childbirth. 2019 Mar 4;19:85. doi: 10.1186/s12884-019-2221-y (PMC6399840; doi:10.1186/s12884-019-2221-y)
Supplement: Supplementary file 2 — Text of the online standardised instruction film for double-layer closure of the uterotomy. The spoken text in the online instruction film, which shows a standardised way to perform double-layer closure of the uterotomy, has been translated into English. (DOCX 15 kb) [file 12884_2019_2221_MOESM2_ESM.docx]

**Additional file 2. Text of the online standardised instruction film for double-layer closure of the uterotomy**

This instruction film is available at [www.2close-trial.nl](http://www.2close-trial.nl)

*Translation from Dutch to English:*

*Welcome to the instruction video regarding ‘double-layer closure’ after a caesarean section, designed for the 2Close study.*

*Step 1. Lateral suture at the surgeons side. The animations were created to explain the real-life video. The lateral suture at the corner of the uterotomy should be placed, as usual, through all layers (serosal, myometrial and endometrial layer) of the uterus, slightly lateral of the incision. Note that one should keep the part of the suture on which the needle is attached long: this part is needed for the second layer.*

*Step 2. Lateral suture at the residents side. Again, slightly lateral of the incision, another lateral suture is placed at the corner of the uterotomy at the other side. This suture should be placed, as usual, through all layers of the uterus.*

*Step 3. Continuous unlocked first-layer of myometrium and endometrium. This is a continuous suture. Insert the needle right under the serosal layer and let it come out deeply, through the endometrium. Insert the needle on the opposite side of the uterotomy (at the same distance) through the endometrium and let it come out right under the serosal layer at the same level at which the needle was inserted at the other side. When you for example inserted the needle half way through the myometrial layer, the needle should come out at the opposite side half way through the myometrial layer.*

*Step 4. To tie the suture of the first layer with the first lateral suture at the surgeons side. Attach the suture of the first layer with the short side of the previously tied lateral suture.*

*Step 5. Second layer (serosal layer), in the direction of the resident. The second layer is also continuous unlocked, which imbricates the first layer. Insert the needle approximately 5mm caudally or cranially of the incision through the serosal layer, and let the needle come out at the same side of the incision, slightly under the serosal layer. You can prevent too much serosal tissue to be included in the suture, when this 5mm distance is followed. The first layer and second layer should be right on top of each other and should be connected closely. Was the first layer a little too deep (not too much myometrial tissue), then the second layer should be placed a little deeper (including a little more myometrial tissue).*

*Step 6. To tie the suture of the second layer with the second lateral suture at the residents side.*

*Additional hemostatic sutures can, of course, be placed when needed. Closure of the peritoneum is not necessary. The fascia should be closed as usual in your hospital. Now, you can see the six steps for double-layer closure in an overview.*

*Thank you very much for watching our instruction video. After watching the video, you should be able to apply a double-layer suture in participants of the 2Close study. We did not create an instruction regarding single-layer closure, since this is done already in 92% of the Dutch gynaecologists. We ask you to do this in a continuous unlocked suture in which you may decide yourself to include or exclude the endometrium in the suture.*
